# Publications in Epidemiology Journals: Japanese perspectives

**DOI:** 10.2188/jea.13.96

**Published:** 2007-11-30

**Authors:** Mahbubur Rahman, Junichi Sakamoto, Tsuguya Fukui

**Affiliations:** 1 Department of Epidemiological and Clinical Research Information Management, Kyoto University Graduate School of Public Health.; 2 Department of General Medicine and Clinical Epidemiology, Kyoto University Graduate School of Medicine.

**Keywords:** epidemiologic research, publication, research productivity, Japan, Medline database

## Abstract

This paper presents information on Japan’s contribution to reputable epidemiologic journals as compared to other leading countries. Ten journals in the field of epidemiology with high impact factors were selected. Articles published in these journals during the period between 1991 and 2000 were searched for the authors’ affiliation using Medline database. We found that Japan contributed only 1.1% of the total number of articles published and ranked 14th among all the countries. The United States of America contributed 56.3% of the total and ranked top followed by the United Kingdom (7.6%), Canada (4.1%), and the Netherlands (3.5%). In a time trend analysis, Japan’s share of articles did not increase significantly in the last decade (p=0.41). Barriers in conducting epidemiologic research in Japan should be identified and appropriate measures should be taken accordingly.

Research in epidemiology has gained tremendous momentum in the last few decades. With vast input from statistics, economics and other social sciences, epidemiology has turned into a very important field of research. Moreover, epidemiologic research designs are of major significance in the context of evidence-based medicine. We conducted this study to quantify Japan’s contribution to selected epidemiology journals in the last decade and to compare it with other leading countries.

## MATERIALS AND METHODS

Ten journals in epidemiology were selected based on their impact factors in the year of 2000 (American Journal of Epidemiology, 3.9; Epidemiology, 3.6; American Journal of Public Health, 3.3; American Journal of Preventive Medicine, 2.2; Journal of Clinical Epidemiology, 2.1; Bulletin of the World Health Organization, 1.9; International Journal of Epidemiology, 1.9; Annals of Epidemiology, 1.8; Journal of Epidemiology and Community Health, 1.8; and Preventive Medicine, 1.6) from a total of 89 journals listed in the category of “Public, Environmental, and Occupational Health” established by the Institute for Scientific Information.^1^ The Medline database was searched in the 1st week of February 2002 to obtain information on the country affiliation for authors of journal articles published during the period from 1991 through 2000 in these 10 journals. Details on methodological procedures have been reported elsewhere.^2^^-^^4^

Nonparametric tests for trend were conducted to elicit any significant change in the contribution of different countries over the time using STATA^®^ statistical software.^5^ All tests of significance were two-tailed and values of p<0.05 were considered significant.

## RESULTS

In total, 14,911 articles were published in the selected epidemiology journals during the period between 1991 and 2000. Affiliation data were available for 14,543 articles (97.5%). Among these, Japan’s contribution amounted to 162 articles (1.1%). Year-wise, Japan’s contribution increased from 0.9% in 1991 to 1.9% in 1999, with a decrease to 1.1% in 2000. This trend was not statistically significant (p=0.41) over the period.

Table 1 shows the 20 top-ranking countries in terms of their volume and share of the total number of articles for each country. The United States of America contributed 56.3% of the total and ranked top among all the countries, followed by the United Kingdom (7.6%), Canada (4.1%), and the Netherlands (3.5%). In a time trend analysis, the share of articles from the USA went down significantly, from 64.1% in 1991 to 56.1% in the year 2000 (p=0.01). Other than the USA, only Italy showed a significant negative trend (p=0.03). On the other hand, Germany (p=0.02), Spain (p=0.01) and Switzerland (p=0.01) showed a significant positive trend.

**Table 1.** Share of articles in epidemiologic research for the top 20 countries

Country	Number of articles published (% of total)		

	1991-2000	1991	2000
	(n=14,911)	(n=1,262)	(n=1,688)
USA ↓	8,395 (56.3)	809 (64.1)	947 (56.1)
UK	1,137 (7.6)	77 (6.1)	147 (8.7)
Canada	606 (4.1)	58 (4.6)	61 (3.6)
Netherlands	527 (3.5)	29 (2.3)	64 (3.8)
Australia	328 (2.2)	16 (1.3)	38 (2.3)
France †	316 (2.1)	21 (1.7)	34 (2.0)
Sweden	302 (2.0)	16 (1.3)	46 (2.7)
Italy † ↓	281 (1.9)	30 (2.4)	28 (1.7)
Finland	261 (1.8)	21 (1.7)	27 (1.6)
Denmark	209 (1.4)	12 (1.0)	23 (1.4)
Switzerland ↑	209 (1.4)	13 (1.0)	35 (2.1)
Spain ↑	209 (1.4)	8 (0.6)	40 (2.4)
Germany ↑	191 (1.3)	6 (0.5)	25 (1.5)
Japan †	162 (1.1)	11 (0.9)	19 (1.1)
Norway †	159 (1.1)	9 (0.7)	15 (0.9)
Israel †	109 (0.7)	9 (0.7)	10 (0.6)
China †	92 (0.6)	10 (0.8)	16 (1.0)
New Zealand †	86 (0.6)	9 (0.7)	9 (0.5)
India †	86 (0.6)	7 (0.6)	9 (0.5)
Brazil †	70 (0.5)	9 (0.7)	5 (0.3)

Ranking based on the total number of articles published during 1991-2000

† The absolute number of articles did not increase significantly over time (1991-2000).

Countries without † sign had significant upward trend regarding absolute number of articles.

↑ Share of articles increased significantly over time.

↓ Share of articles decreased significantly over time.

Data did not sum up to 100 percent because shares of other countries are not included.

Data of China also include Hong Kong’s share.

## DISCUSSION

Japan’s contribution to epidemiology journals (1.1% of the total) is comparable to its contribution to highly reputable general medical journals (0.7% of the total).^6^ Its position was also at the bottom of the developed countries in terms of conducting randomized controlled trials.^7^ Thus, it is undeniable that Japan lags behind other developed countries in conducting research on general medicine (0.7% of total)^6^ and epidemiology (1.1% of total), although it is doing better in basic science (3.1% of the total),^6^ nuclear medicine (11.4% of total),^2^ and orthopedics (8.3% of total).^3^ In terms of the proportion of Japan’s contribution to basic science, general medicine, nuclear medicine, and orthopedics Japan ranked 4th, 14th, 2nd and 3rd in the world, respectively,^2^^-^^4^ although it is ranked 2nd in the world in terms of total number of articles in the biomedical field.^9^ If we consider the size of the Japanese economy (2nd in the world) and per capita gross national product (32,030 US$, 7th in the world),^8^ Japan’s contribution to epidemiology journals is both poor and clearly unsatisfactory.

The following measures could be helpful in boosting epidemiologic research in Japan. First, a separate pool of faculties for epidemiologic research is needed at every health-care institution. Second, medical students, residents, and postgraduate students should be exposed to appropriate research methodology through a well-designed curriculum. It is also crucial to equip general practitioners with the necessary tools to understand study design, conduct research, analyze and interpret data, and critically evaluate published literature. As a result, they would be in a position to conduct epidemiologic research and to cooperate with other researchers. Funding agencies would also have to encourage them toward research activities by allocating funds. A reasonable amount of funds should also be directed to epidemiology-related departments for conducting both patient-oriented (clinical epidemiology) and population-oriented (field epidemiology) researches. Third, large cohort studies and randomized controlled trials, which are reported to be limited in Japan, should be initiated by policy makers.

There is no doubt that the USA is on top in every field of medical research.^2^^-^^4^^,^^7^^,^^9^^,^^10^ However, its share of epidemiologic research articles went down in the last decade. This might be attributed to the following situations. First, the USA conducted great deal of research on epidemiology but it is now changing focus to newer avenues such as methods of eliciting new risk factors for health and health-related events and of finding innovative ways for disease prevention and so the share might have gone down due to “overkill”. Second, other countries have been catching up and increasing their share of epidemiologic research. A similar downward trend has also been observed in the USA in respect to research articles in basic and clinical science and nuclear medicine.^2^^,^^4^ The results of our study also revealed that among developed countries with smaller economies, such as Australia, Denmark, Finland, the Netherlands and Switzerland, made a greater contribution than countries with larger economies such as Japan and Germany. More researches are necessary to elucidate the factors responsible for the meager number of publications on epidemiology from these two countries.

There are some limitations in this study. First, some studies were collaborative efforts conducted by mixed teams of local and international researchers and only the communicating authors’ affiliations were included as the origin of research in the Medline database. Second, some articles on epidemiology might have been published in clinical journals because of their clinical relevance or in other epidemiologic journals with a lower impact factor or in other interdisciplinary journals. If these factors are taken into account, the actual number of research papers for each country would be higher than the results we obtained. However, this situation is not confined to a particular country, but rather related to all countries. Thus, the absolute number of articles shown here does not reflect the true situation in terms of all epidemiology journals, but our estimates on the proportion of the contributions made are probably not far from the real situation. Third, we selected 10 journals according to their impact factors. This was an arbitrary number. Inclusion of more journals might change the share of contribution. For example, including Journal of Epidemiology, the official journal of Japan Epidemiologic Association, in our analysis, could increase Japan’s contribution as a whole. In that case, however, we had to include many more journals. However, a milestone in epidemiologic research in Japan is the Journal of Epidemiology, which has been publishing for the last 12 years and certainly exerting positive influence for high quality research in this important field.

Our analysis revealed that Japan’s contribution to epidemiology journals is not up to expectation. Ranking in 14th place and showing no significant positive trend over time implies that a major initiative from leading epidemiologists and government agencies is needed to create positive change in this sector.
